# Predicting phenotypes of beef eating quality traits

**DOI:** 10.3389/fgene.2023.1089490

**Published:** 2023-02-01

**Authors:** Mehrnush Forutan, Andrew Lynn, Hassan Aliloo, Samuel A. Clark, Peter McGilchrist, Rod Polkinghorne, Ben J. Hayes

**Affiliations:** ^1^ Queensland Alliance for Agriculture and Food Innovation, The University of Queensland, Brisbane, QLD, Australia; ^2^ School of Environmental and Rural Science, University of New England, Armidale, NSW, Australia; ^3^ Birkenwood International, Hawthorn, VIC, Australia

**Keywords:** BayesR, beef cattle, eating quality, high-density SNP, phenotype prediction

## Abstract

**Introduction:** Phenotype predictions of beef eating quality for individual animals could be used to allocate animals to longer and more expensive feeding regimes as they enter the feedlot if they are predicted to have higher eating quality, and to sort carcasses into consumer or market value categories. Phenotype predictions can include genetic effects (breed effects, heterosis and breeding value), predicted from genetic markers, as well as fixed effects such as days aged and carcass weight, hump height, ossification, and hormone growth promotant (HGP) status.

**Methods:** Here we assessed accuracy of phenotype predictions for five eating quality traits (tenderness, juiciness, flavour, overall liking and MQ4) in striploins from 1701 animals from a wide variety of backgrounds, including *Bos indicus* and *Bos taurus* breeds, using genotypes and simple fixed effects including days aged and carcass weight. The genetic components were predicted based on 709k single nucleotide polymorphism (SNP) using BayesR model, which assumes some markers may have a moderate to large effect. Fixed effects in the prediction included principal components of the genomic relationship matrix, to account for breed effects, heterosis, days aged and carcass weight.

**Results and Discussion:** A model which allowed breed effects to be captured in the SNP effects (e.g., not explicitly fitting these effects) tended to have slightly higher accuracies (0.43–0.50) compared to when these effects were explicitly fitted as fixed effects (0.42–0.49), perhaps because breed effects when explicitly fitted were estimated with more error than when incorporated into the (random) SNP effects. Adding estimates of effects of days aged and carcass weight did not increase the accuracy of phenotype predictions in this particular analysis. The accuracy of phenotype prediction for beef eating quality traits was sufficiently high that such predictions could be useful in predicting eating quality from DNA samples taken from an animal/carcass as it enters the processing plant, to enable optimal supply chain value extraction by sorting product into markets with different quality. The BayesR predictions identified several novel genes potentially associated with beef eating quality.

## Introduction

Historically the value of breeding cattle has been principally based on improved growth, milk and fertility traits and correlated measures of meat quality and quantity. Recent years have seen a shift from producer-driven to consumer-driven beef production, with significant emphasis placed on consumer satisfaction and interest in eating quality indicators, with incentives implemented by beef brands for improved compliance and product quality.

Genomic selection (Meuwissen et al., 2001) offers the opportunity to select directly for beef eating quality. Samples of beef that are consumer evaluated for quality can be genotyped for genome wide markers and then marker prediction equations for eating quality derived. Then young bulls and heifers could be genotyped and evaluated on their eating quality genomic estimated breeding values (GEBVs) (Pimentel and König, 2012; Bedhane et al., 2019; Magalhães et al., 2019).

Phenotype predictions, which predict the eating quality of beef from an individual animal, may also be of interest, for example to sort carcasses into consumer or market value categories, or to allocate animals to longer and more expensive feeding regimes as they enter the feedlot if they are predicted to have higher eating quality. Phenotype predictions could include genetic effects, predicted from genetic markers, as well as estimates of fixed effects such as days aged and carcass weight. Alsahaf et al. (2018) used an analogous approach to predict slaughter age in pigs and found combining estimates of fixed effect predictors and genetic effects gave higher accuracy than genetic effects alone.

Our aim here was to determine the most accurate method for predicting phenotypes of beef eating quality traits from genotypes and other independent factors such as carcass weight and days aged, using consumer eating quality data from the striploins of 1701 beef cattle.

## Materials and methods

The phenotype data consisted of eating quality traits from striploin samples from 1701 cattle collected from 65 cohorts between 1997 and 2019 in Australia. The eating quality traits were scores for tenderness (TENDER), juiciness (JUICY), flavour (FLAVOR), overall liking (OVERALL), and MQ4 score formed by weighting the four sensory scores, MQ4 = 0.4 × TENDER + 0.1 × JUICY + 0.2 × FLAVOR + 0.3 × OVERALL, obtained using optimum linear discriminating function (Watson et al., 2008). All samples were assessed by consumer panels as described by Watson et al. (2008). Each sample was 70 mm long by 40 mm wide by 25 mm thick, cooked for 5 min 15 s on a silex grill with the top plate being 195 Celsius and bottom is 210 Celsius, rested for 3 min, cut in half and served directly to consumers who are instructed to eat it immediately after receiving. Each panel assessment of an animal was eaten by 10 consumers, to remove extreme values, the two highest and two lowest values were removed. The remaining six values were then averaged for a “clipped” sensory score (as described by Watson et al., 2008). Each treatment group within an experiment is a cohort. All animals in the project were HGP free.

The breed background of the animals was diverse, including 261 Brahman (*Bos indicus*), 285 Angus, 274 Hereford, 38 Shorthorn, 72 Holstein, 23 Jersey (*Bos taurus*), 100 Belmont Red, 83 Santa Gertrudis (composite), 121 crossbred and 444 with unknown breed. The animals included 1,319, 345 and 37 steers, heifers, and bulls, respectively, although sex was completely confounded with contemporary group. As these were largely commercial animals, little pedigree was available, however breed information could be reconstructed by genotype as described below.

Genotypes with a missing rate >0.1, a minor allele frequency (MAF) of <0.01 and those departing from the Hardy-Weinberg equilibrium at *p* < 1 × 10^−8^ were removed. After quality control, the genotypes were imputed up to 7,09,068 SNPs (Illumina HD array) using findhap4 (VanRaden et al., 2013). The reference panel for imputation was 4,506 animals genotyped with the Illumina HD array of a wide variety of breeds and crossbreds, including *Bos indicus* breeds, *Bos taurus* breeds, and crossbreds and composites, encompassing the target breeds in this study. All imputation achieved with 91%–98% accuracy for the current study. The first four principal components (PCs) of the genomic relationship matrix (derived with GCTA, Yang et al., 2011) which comprised 25% of the variance in genotype data were used as proxies for breed composition. Inspection showed that the first principal component was 99.9% correlated with *Bos indicus* content.

The BayesR approach (Erbe et al., 2012) with four strategies were implemented for phenotype predictions. In the BayesR, the variance associated with the *i*th SNP is assumed to come from one of four distributions $\sigma_{i}^{2}=\left\{0,10^{-4}\sigma_{g}^{2},10^{-3}\sigma_{g}^{2},{10^{-2}\sigma }_{g}^{2}\right\}$ **,** where $\sigma_{g}^{2}$ is the genetic variance of the trait. This allows the BayesR model (Moser et al., 2015) to have a flexible SNP effect distribution which is a mixture of four possible normal distributions: $\left.N\left(0,0\right),N\left(0,10^{-4}\sigma_{g}^{2}\right),{N(0,10}^{-3}\sigma_{g}^{2}),{N(0,10^{-2}\sigma }_{g}^{2}\right)$ **,** all with a mean of 0 but with different variances.

In strategy 1, the accuracy of predicting phenotype from genomic breeding values only was assessed. A mixed linear model was fitted, including fixed effects of PCs and heterosis, for each of the 5 eating quality traits separately using BayesR (Erbe et al., 2012). The heterosis was defined as the regression of the trait on proportion of heterozygote loci across all loci for each animal.

y=µ+cg+days_aged+carcass_weight+PC1+PC2+PC3+PC4+heterosis+Zg+e, (1)

where **y** is phenotype; **cg** is a fixed contemporary group effect (65 groups), **days_aged** is a covariate of days aged after slaughter (ranging from 3 to 35 days; mean ± SD: 10.92 ± 5.18 days), **carcass_weight** is a covariate on carcass weight (ranging from 50.6 to 576 kg; mean ± SD: 261 ± 74 Kg), **PC1** to **PC4** are the first 4 principal components of the genomic relationship matrix, fitted to control for *Bos indicus* content and breed, ***heterosis*** is a regression on marker heterozygosity, ***Z*** is matrix allocating genotypes of individuals to SNP effects, ***g*** is a vector of SNP effects, and ***e*** is a random residual with $e∼N\left(0,\sigma_{e}^{2}\right)$ .

Next, phenotype was predicted with a genomic estimated breeding value in a validation set as

$$\hat{y}=Z\hat{g}$$ (2)

In strategy 2, phenotype predictions were made first by fitting the model 1 to the training set like strategy 1, but in a validation set phenotype was predicted including all estimates of fixed effects as below:

$$\hat{y}=Z\hat{g}+\hat{days\_aged}+\hat{carcass\_weight}+\hat{PC1}+\hat{PC2}+\hat{PC3}+\hat{PC4}+\hat{heterosis}$$ (3)

where for example $\hat{days\_aged}$ is the number of days aged for the sample in the validation set multiplied by the estimate of the effect of ***days_aged*** from model 1.

In strategy 3, exactly the same model as strategy 1 and 2 (Model 1) were used for the training set, however for a validation set, the phenotype was predicted only from genetic effects and fixed effects that were derived from genotypes (i.e., those effects that would be available before the animal entered the processing plant, in the feedlot for example) as below:

$$\hat{y}=Z\hat{g}+\hat{PC1}+\hat{PC2}+\hat{PC3}+\hat{PC4}+\hat{heterosis}$$ (4)

Finally, in strategy 4, model 5 was implemented in training set as below:

y=µ+cg+days_aged+carcass_weight+Zg+e (5)

where **y**, **cg**, ***days_aged***, ***carcass_weight***, **Z**, **g**, and **e** are the same as Model 1.

The assumption here is that all genetic effects, including breed effects, are captured by the SNP effects when PCs are not explicitly fitted. Next, phenotype was predicted with a genomic estimated breeding value in a validation set using Model 2.

To evaluate accuracies of phenotype prediction from the 4 strategies, five-fold cross-validation was used, with random grouping of animals such that all groups have approximately equal size. In each rotation of the cross validation the phenotypes of 1 group were masked and the remaining 4 groups were used to estimate the GEBV of the group without phenotypes. The accuracy of phenotype prediction for each group was calculated as the Pearson correlation between predictions and raw phenotypes of animals for which their phenotypes were masked. Correlations were averaged across 5 groups and standard error (SE) was calculated as the standard deviation divided by the square root of the number of groups i.e., $SE=\frac{STD}{\sqrt{5}}$ .

For each cross-validation fold, the BayesR model simultaneously provides estimates for the SNP effects ( $\hat{g})$ , the genetic $\hat{\sigma_{g}^{2}}$ and residual variances $\hat{\sigma_{e}^{2}}$ and the SNP-basedt heritability $h^{2}=\frac{\hat{\sigma_{g}^{2}}}{\hat{\sigma_{g}^{2}}+\hat{\sigma_{e}^{2}}}$ . These estimates were mean of the posterior distribution for each parameter. The posterior distributions were sampled using Markov Chain Monte Carlo (MCMC) with Gibbs Sampling in GCTB (Zeng et al., 2018) with 25,000 iterations of which the first 5,000 are discarded as burn-in and thinned by 10 (2,000 MCMC samples).

We also investigated the posterior probability of inclusion of each SNP in strategy 1, to identify any genes of moderate effect on eating quality. Note that the posterior probabilities of inclusion of SNP from the other strategies were very similar to that in strategy 1 (Supplementary File S1).

We also identified the nearest genes within a window of 1 Mb upstream or down stream of top 20 SNPs with highest posterior probability of inclusion for all five eating quality traits. We then performed Gene Ontology analysis on the list of genes associated with eating quality traits using the DAVID web server (Huang et al., 2009) and considering the entire *Bos taurus* gene set as a reference data set.

## Results

Heritabilities of the eating quality traits from Model 1 ranged from moderate (0.37) to low (0.23), Table 1. The highest and lowest heritability was observed for TENDER and JUICY, respectively.

**TABLE 1:** Means (±standard deviations), heritability (±SE), and accuracy of predicting phenotype (±SE) of phenotype prediction for 5 eating quality traits [tenderness (TENDER), juiciness (JUICY), flavour (FLAVOR), overall liking (OVERALL), and MQ4 score formed by weighting the four sensory scores]. See methods for description of strategies. The significant difference of strategies was shown in different letters (a, b, c).

Trait	Means	h^2^	Accuracy			
			Strategy 1	Strategy 2	Strategy 3	Strategy 4
TENDER	56.84 ± 16.99	0.37 ± 0.05	0.20 ± 0.02^a^	0.40 ± 0.03^b^	0.49 ± 0.02^c^	0.50 ± 0.02^c^
JUICY	57.53 ± 14.47	0.23 ± 0.05	0.08 ± 0.02^a^	0.30 ± 0.01^b^	0.41 ± 0.02^c^	0.43 ± 0.01^c^
FLAVOR	58.83 ± 12.52	0.30 ± 0.04	0.16 ± 0.02^a^	0.34 ± 0.02^b^	0.42 ± 0.02^c^	0.43 ± 0.02^c^
OVERALL	57.94 ± 14.52	0.30 ± 0.04	0.16 ± 0.03^a^	0.35 ± 0.02^b^	0.45 ± 0.02^c^	0.47 ± 0.01^c^
MQ4	47.46 ± 14.10	0.32 ± 0.04	0.21 ± 0.02^a^	0.37 ± 0.02^b^	0.47 ± 0.02^c^	0.49 ± 0.01^c^

The predictions of eating quality phenotype based on GEBV alone (Strategy 1) was modest, Table 1. When breed effects and heterosis were not explicitly fitted in the model in the training set, that is they were included in the SNP effects, accuracy of phenotype predictions was much higher (Strategy 4). Interestingly, adding the estimated effect of carcass weight and days aged did not improve the accuracy of phenotype prediction (Strategy 2). The accuracy of phenotype prediction when Strategy 3 was implemented (only including fixed effects that could be derived from genotypes) were slightly worse than implementing Strategy 4 for all traits.

We conducted a follow-up study of potential genes affecting eating quality in cattle. We used the nearest genes within a window of 1 Mb upstream or down stream of top 20 SNPs with highest posterior probability of inclusion for all five eating quality traits. The most strongly enriched pathway identified by Gene Ontology analysis of genes associated with eating quality was “dopamine metabolic process” (*p-value* < 8.7 × 10^−5^).

**FIGURE 1:**
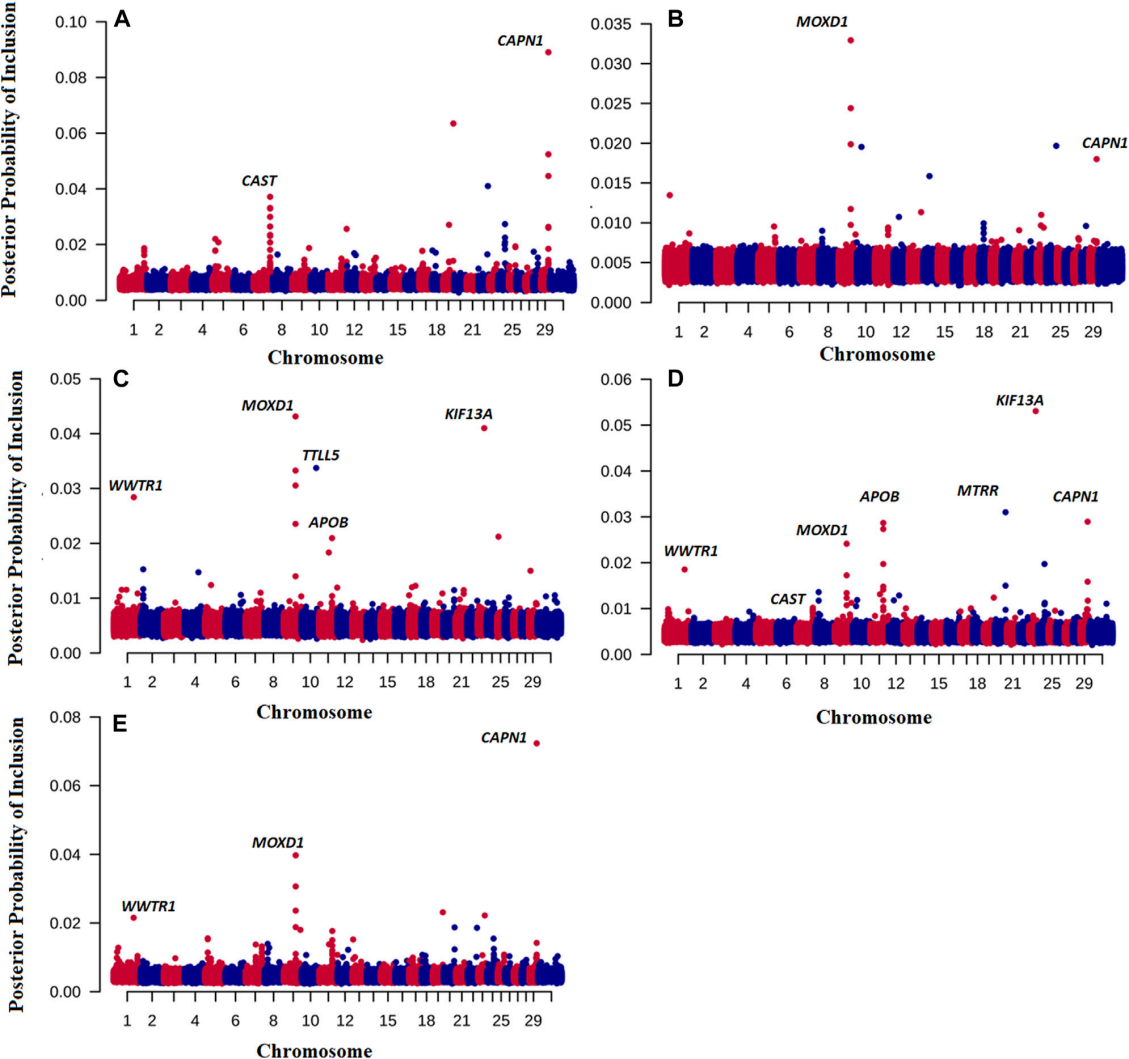
Posterior probability of inclusion in the BayesR prediction model for 7,09,698 SNP for TENDER **(A)**, Juicy **(B)**, Flavour **(C)**, OVERALL **(D)**, and MQ4 **(E)** traits. Odd chromosomes are colored in red, even chromosomes are colored in blue.

Of note is that we have also discovered novel genes associated with the traits of interested by running the BayesR model. Figure 1 shows the Manhattan plot of probability a SNP is included in the BayesR prediction model for all traits. Interestingly, for TENDER and MQ4, SNPs in and close to the *μ-calpain* (*CAPN1*) and *Calpastatin* (*CAST*) genes had the highest probability of inclusion in the model, (Figures 1A, E). For JUICY and FALVOR the monooxygenase DBH like 1 (*MOXD1*) had the highest probability of inclusion. However, the same SNP close to the *MOXD1* were also included in the prediction model for OVERALL and MQ4 (Figures 1D, E). For OVERALL, SNPs associated with *CAST (Calpastatin), CAPN1*, Kinesin Family 13A (*KIF13A*) and Apolipoprotein B (*APOB*) genes were also included in the prediction model (Figure 1D). The same SNPs close to *KIF13A* and *APOB* had the highest posterior probability of inclusion for FLAVOR as well (Figure 1C).

## Discussion

Generally, levels of heritability in this study (0.23–0.37; Table1) were in agreement with previous studies (Kause et al., 2015; Wolcott et al., 2009), showing the possibility of improvement of these traits by conducting selection. For example, Kause et al. (2015) reported the moderate heritability for carcass weight (0.39–0.48) in five beef cattle breeds in Finland (Hereford, Aberdeen Angus, Simmental, Charolais and Limousin).

**FIGURE 2:**
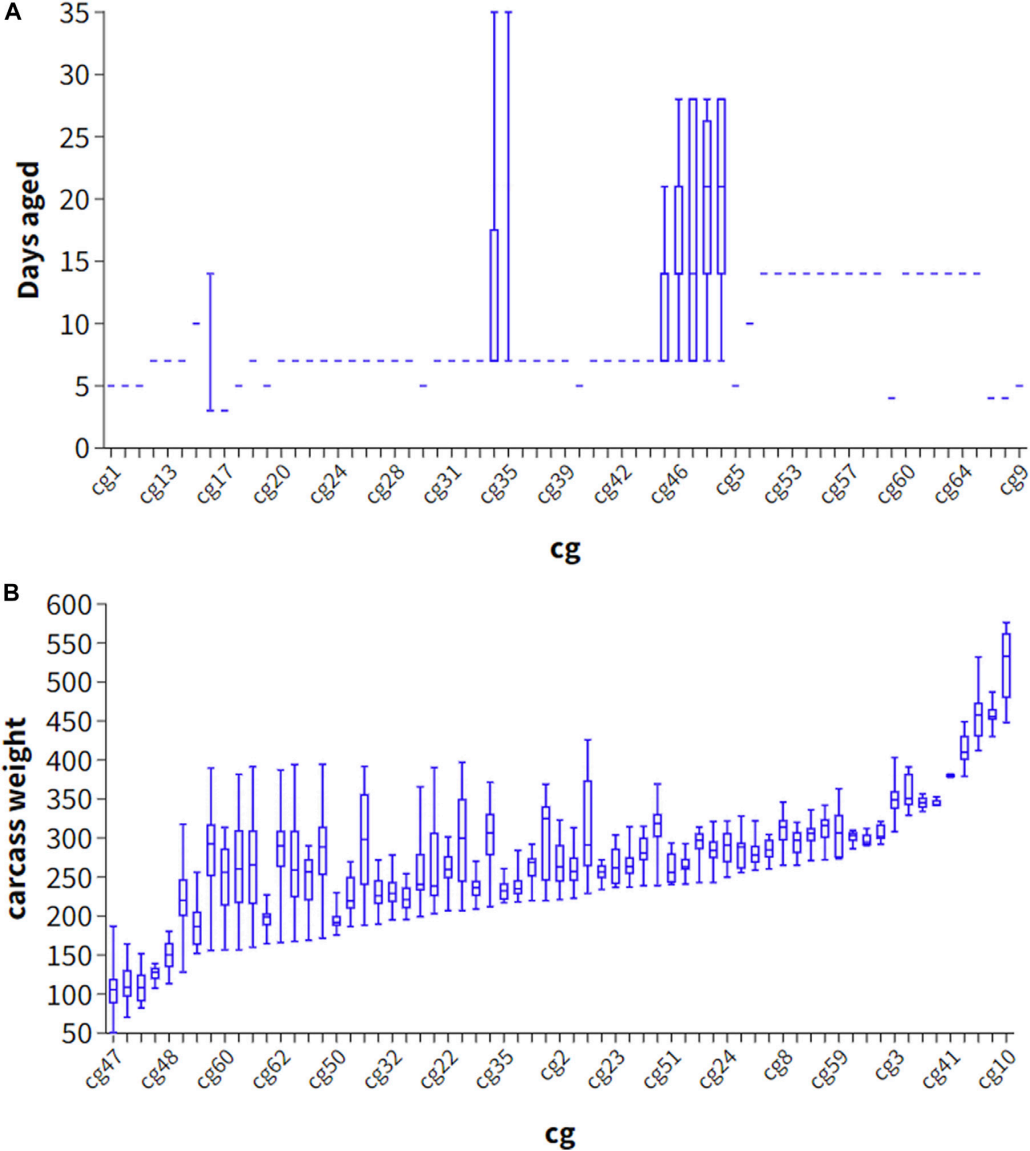
Scatter plot of contemporary group (cg) and days aged **(A)** and carcass weight **(B)** in 1701 striploin samples.

Phenotype predictions, which predict the eating quality of beef from an individual animal, may benefit industry by sorting carcasses into consumer or market value categories. Phenotype can be predicted by including the genetic effects such as breed, heterosis and breeding value effects, as well as fixed effects such as days aged and carcass weight, hump height and ossification and hormone growth promotant (HGP) status. Interestingly, adding the estimated effect of carcass weight and days aged did not improve the accuracy of phenotype prediction (Strategy 2), likely because these effects were relatively poorly estimated in our study due to the large number of cg groups and confounding of these effects with the cg group effect (Figures 2A, B). Explicitly fitting breed effects (as PCs) and heterosis as covariates and using these estimates in the prediction (Strategy 3), performed slightly worse than not explicitly fitting these effects, and allowing these effects to be captured in the SNP effects (Strategy 4) perhaps because breed effects when explicitly fitted were estimated with more error than when incorporated into the (random) SNP effects.

For TENDER, the *CAST* and *CAPN1* genes had the highest probability of inclusion in the model (Figures 1A–E), that is SNP associated with both these genes were used in the prediction model. Considerable evidence has demonstrated that the *CAPN1* gene and its inhibitor *CAST* gene are major factors affecting meat quality (*e.g*., (Sun et al., 2018)). These results shed some light on why our phenotype predictions including breed effects (e.g., Strategies 2,3,4) are so much more accurate than predictions based on GEBV alone - part of the prediction accuracy is derived from predicting the *CAST* effect, which is at quite different frequencies in *Bos indicus* and *Bos taurus* cattle (Page et al., 2002; Casas et al., 2006), and therefore partially captured by PC1. In addition to the *CAST* effect. *Bos indicus* cattle have been widely reported to have less tender meat, and hump height is currently used as proxy for *Bos indicus* content when predicting Meat Standards Australia grade (Watson et al., 2008). It is important to point out that our eating quality predictions are relevant for mixed cohorts including *Bos indicus* and *Bos taurus* cattle and their crosses. If predictions were made for single breed cohorts (with no variation in *Bos indicus* content), the predictive information from PC1 would be irrelevant, and the prediction accuracy would default to that from the GEBV alone (e.g., Strategy 1).

The results implicate some interesting candidate genes for eating quality. Kinesin Family 13A (*KIF13A*) is in a pathway associated with skeletal muscle cells increasing insulin signalling, glucose uptake, and maximal oxygen consumption (Massart et al., 2021). Apolipoprotein B (*APOB*) is a building block of a type of lipoprotein called a chylomicron. As food is digested, chylomicrons form to carry fat and cholesterol from the intestine into the bloodstream.

In conclusion, the accuracy of phenotype prediction for beef eating quality traits was sufficiently high that such predictions could be useful in predicting eating quality from samples taken from an animal/carcass as it enters the processing plant, to sort for markets with different quality. The BayesR predictions identified several novel genes potentially associated with beef eating quality. Future predictions will be expanded to incorporate all the parameters in the Meat Standards Australia (MSA) models (Watson et al., 2008) as well as genotype information.

## Data Availability

The original contributions presented in the study are included in the article/Supplementary Material, further inquiries can be directed to the corresponding author.

## Abbreviations

HGP, hormone growth promotant; SNP, single nucleotide polymorphism; GEBV, genomic estimated breeding value; TENDER, tenderness; JUICY, juiciness; FLAVOR, flavour; OVERALL, overall liking; PC, principal component; SE, standard error; SD, standard deviation; MCMC, Markov Chain Monte Carlo; MSA, Meat Standards Australia.
